# Supplementary material for: Chromothripsis during telomere crisis is independent of NHEJ, and consistent with a replicative origin
Source: Genome Res. 2019 May;29(5):737–49. doi: 10.1101/gr.240705.118 (PMC6499312; doi:10.1101/gr.240705.118)
Supplement: Supplemental Material [file supp_gr.240705.118_Supplemental_file_1.zip › contigs/annotated_contigs/DB57/contig.2.DB57_length_609_mean_cov_11.5123152709.docx]

**DB57_length_609_mean_cov_11.5123152709**

```
CCTTAATGTCTTTTTCTTTCAGATTAAAAACTTACTTAAGGAAAGAGCTGGTATGTATGAAATCCCTCGGGTTTTGTTTGCCTGGGGAA
 >chr11:49722759-49723113 + E=3e-183
TTTTTTTATTTCTCCTTCATGTCTGCAGGATATTTTCACTGGATATACTATTCTTGGATAAAAGATTTTTTTGTTTGGTTTGTTTTTGG

TTTTGTTTTTGTTTTCCCTTCAGCACTTTAGATATGCCATGCCACTCTCTCCTGGCGTGTAAGGTCAAGGCTTTAAGTCTGCTGCCAGA

TGTATTGGTGCTCCTTTGTATGTTATTTGTTGCTTTTTTTCTTGCTGCCTTTATGATCCTTTCTTTATTTTTGATGTTTGGGAGTTT|A

T|TAGCCAGTTGCCACATTTAAGTGGGTGAGAAGGGAGGCTGGTGAGAAGTTTCTTTTTTATTTTATTAAGTTTCAGGGTACATGTGCA
  >chr5:83594253-83594506 - E=2e-140
GGATTTGCAGGTTTGTTACATAGGTAAACGTGTGCCATGGTGGTTTACTGCACCTATCAACCTATCACCTTGGTATTAAGCCCAGTATG

CATTAGCTGTTTTTCCTGATGCTCTCTGCCAAGCCTCTGACAGGCCCCAGTGTGTGTTGTTCCCCTCCCCGTGTCCA
```
